# Crystal Methamphetamine Use in Sexual Settings Among German Men Who Have Sex With Men

**DOI:** 10.3389/fpsyt.2019.00886

**Published:** 2019-12-06

**Authors:** Henrike Schecke, Toby Lea, Annette Bohn, Thorsten Köhler, Dirk Sander, Norbert Scherbaum, Daniel Deimel

**Affiliations:** ^1^Department of Psychiatry and Psychotherapy, University of Duisburg-Essen, LVR-Hospital Essen, Essen, Germany; ^2^Catholic University of Applied Sciences, German Institute for Addiction and Prevention Research, Köln, Germany; ^3^Centre for Social Research in Health, UNSW Sydney, Sydney, NSW, Australia; ^4^Catholic University of Applied Sciences, German Institute for Addiction and Prevention Research, Aachen, Germany; ^5^Deutsche AIDS-Hilfe e.V., Berlin, Germany

**Keywords:** methamphetamine, men who have sex with men, mental health, harm reduction, HIV, chemsex

## Abstract

**Introduction:** Men who have sex with men (MSM) are a vulnerable subgroup for problems with substance use, including crystal methamphetamine. Drug use in sexual settings, commonly referred to as “chemsex,” has been an issue of growing concern in MSM communities. Recreational drugs commonly associated with chemsex include crystal methamphetamine, gamma-hydroxybutyrate/gamma-butyrolactone (GHB/GBL), mephedrone, and ketamine. Drug use in sexual settings is correlated with sexual practices associated with the acquisition and transmission of sexually transmitted infections, including HIV and hepatitis C. Adverse mental health outcomes are often reported at higher rates among MSM who use methamphetamine.

**Methods:** This paper refers to a subset of participants from the *German Chemsex Survey*, an MSM-community recruited, self-completed online survey with a self-selected convenience sample. Participants who used crystal methamphetamine for sex (n = 130) were compared to participants who did not use drugs for sex (n = 177). The survey comprised 420 different items considering recreational substance use, substance use in sexual settings, harm reduction strategies, mental health, sexual transmitted infections, and mental health care service utilization.

**Results:** A total of 1,583 men started the survey; 1,050 participants provided information on substance use. Twenty-seven percent of participants used crystal methamphetamine in the last 12 months, and of those, 89% used methamphetamine in a sexual setting and 50% reported injecting methamphetamine. Regarding mental health, participants who reported methamphetamine use in sexual settings were more likely to report symptoms of depression, somatization, anxiety, and posttraumatic stress disorder (PTSD) than the German male general population. Participants who reported methamphetamine use for sex were more likely to report symptoms of major depression, being HIV positive, and taking HIV pre-exposure prophylaxis (PrEP) than participants who did not report methamphetamine use. Most participants used harm reduction practices to reduce the risks associated with using methamphetamine in sexual settings.

**Conclusion:** Crystal methamphetamine is used in the context of sexual activities by German MSM. Poorer mental health status than in the male general population was observed. MSM who used methamphetamine in this study seemed to be aware of potential health risks associated with their substance use and utilized harm reduction strategies and biomedical HIV prevention strategies like PrEP.

## Introduction

Men who have sex with men (MSM) are a vulnerable subgroup for problems with substance use, including crystal methamphetamine (1, 2). Methamphetamine use in MSM populations is a growing issue of concern globally. Numerous studies from the United States (3), diverse countries in Asia (4), the United Kingdom (5–7), Ireland (8), Australia (9, 10), and the European Union (11) have consistently reported a heightened prevalence of methamphetamine use among MSM compared to heterosexual men.

Drug use in sexual settings, now commonly referred to as "chemsex," has been an issue of growing concern in MSM communities in recent years (5). Recreational drugs commonly associated with chemsex include crystal methamphetamine, gamma-hydroxybutyrate/gamma-butyrolactone (GHB/GBL), mephedrone, and ketamine, and are typically used with the intention to enhance, intensify, and prolong sexual experiences (2). Drug use in sexual settings can have benefits for MSM, such as fostering social and sexual connections with other men and the exploration of sexual desires (12). However, drug use in sexual settings is also associated with the acquisition and transmission of sexually transmitted infections (STIs), including HIV (13). It has been consistently shown that MSM who use drugs for sex are more likely to be HIV positive (14, 15) and have higher rates of STIs and hepatitis C (HCV) (16, 17) than those who do not engage in these practices. HIV-positive MSM are more likely to initiate methamphetamine use after seroconversion, for some men as a coping strategy (18). Current methamphetamine use also negatively affects adherence to the antiretroviral therapy (ART) in HIV-positive MSM (19).

Research indicates that injecting methamphetamine in sexual settings ("slamming") is also common in some networks of MSM (20, 5). Injecting drugs potentiates health risks such as blood-borne infections like HCV and HIV, injecting-related injuries and infections, overdose, and more severe substance dependence (21). Studies show that HCV infections are increasing among MSM, in particular among HIV-positive MSM who have never injected drugs (22–24). Drug use in sexual settings is also associated with engagement in group sex, having multiple sex partners (25, 26), transactional sex, sharing sex toys, sex practices with risks for injuries (27), and condomless anal intercourse (28) which also increases STI risks.

Adverse mental health outcomes are also reported at higher rates among MSM with longer-term methamphetamine use. In an US sample of ethnic minority MSM who use methamphetamine, a higher prevalence of major depressive disorder, social phobia, obsessive–compulsive disorder, antisocial personality disorder, and posttraumatic stress disorder (PTSD) and a higher risk for suicide attempts was reported, compared to the male US general population. In addition, mental health disorders were more commonly reported among men with more severe methamphetamine use disorders (29, 30). Lopez-Patton et al. found significantly higher rates for major depression and childhood trauma among methamphetamine-using MSM (31). In an online cohort study in Australia, while methamphetamine use overall was not negatively associated with mental health, men who were methamphetamine dependent were more likely to report depression and anxiety than men who used methamphetamine but were not dependent (32).

Crystal methamphetamine use is uncommon in most parts of Germany, with exceptions to regions near the Czech border (33). In a representative survey of the general population in Germany, the 12-month prevalence of methamphetamine use was only 0.2% among men (34). To date, there has been limited research about methamphetamine use among MSM in Germany. A recent study on motivations for psychostimulant use among German adults found that MSM most commonly reported using methamphetamine in sexual settings (35). However, little is known about German MSM who use crystal methamphetamine in sexual settings, nor their mental health (e.g., depression, anxiety, and posttraumatic stress), HIV prevention strategies such as pre-exposure prophylaxis (PrEP), or drug-related harm reduction practices. This paper aims to address these research gaps using findings from a recent national online survey. In addition, the paper examines the utilization of mental health, alcohol, and drug treatment and related support services among German MSM.

## Methods

### Sample

The analysis refers to a subset of participants from the *German Chemsex Survey*, an MSM-community recruited, self-completed online survey with a self-selected convenience sample. Eligible participants were at least 18 years of age, identified as male, and as gay, bisexual, or MSM. The present study is focused on two groups: men who reported crystal methamphetamine use in sexual settings in the previous 12 months, and men who reported no illicit drug use in sexual settings in the previous 12 months.

The survey was promoted *via* Lesbian, Gay, Bisexual, Transexual, Intersexual and Queer (LGBTIQ)-community websites, social media postings, HIV non-profit organizations, free-of-charge advertisements on "planetromeo" (MSM-dating website/smartphone application), and HIV/sexual health care service providers. The survey was online for 12 weeks between September and December 2018 and used the open-source survey software "LimeSurvey." All data collected were anonymous. Participants could skip questions they did not want to answer and could withdraw from the survey at any time during completion. At the end of the survey, links to nationwide accessible psychosocial support services were presented to offer support for participants who felt uncomfortable as a consequence of being confronted with questions on substance use and mental health issues. Ethical approval for the study was received from the Ethics Committee of the Medical Department of the University of Duisburg-Essen (UDE-18-8209-BO).

### Measures

The survey consisted of 420 items including demographic characteristics, recreational substance use, substance use in sexual settings, mental health, sexual behavior, STIs, psychosocial/health outcomes of methamphetamine use, harm reduction practices, and use of mental health care and drug treatment services. Mental health was assessed using the German version of the Patient Health Questionnaire (PHQ-D) with subscales for depressive symptoms (PHQ-9), generalized anxiety symptoms (GAD-7), and somatization symptoms (PHQ-15) (36). The PHQ-9 scale assesses severity of depressive symptoms with a maximum score of 27. The PHQ-15 score gives information about symptoms of somatization with a maximum value of 30. GAD-7 measures symptoms of anxiety with a maximum of 21. A score of 10 or above on each of the three scales signifies an at least moderate major depressive episode, moderate levels of somatization, and moderate levels of clinical anxiety. For trauma and PTSD, the life events checklist for *DSM-5* (37) as well as the four-item PTSD primary care screener (38) were conducted.

### Statistical Analysis

Given that participants were able to stop and save their data at any point of the survey and the survey software was not programmed in a "forced choice" format, sample size varies for different items. Data analysis was conducted using IBM SPSS Statistics 25.0. For group comparisons of participants who reported methamphetamine in sexual settings (methamphetamine group) with participants who did not report drug use in sexual settings (no drug use for sex = NDUS group), Chi² tests were used for binary and categorical dependent variables and Mann–Whitney U tests for continuous dependent variables which were not normally distributed. Where statistical tests were performed, p-values of <0.05 were taken to be statistically significant.

## Results

### Sample

A total of 1,583 men started the survey, and 1,050 participants provided information on substance use (66.3%). Of the 1,050 participants who provided data on substance use, 231 (22%) reported any methamphetamine use: 36.8% ever, 26.8% in the last 12 months, 17.3% in the last 30 days, and 19% in the last 7 days. Fifty percent of the methamphetamine group had injected methamphetamines in the last 12 months. Methamphetamine use in a sexual setting in the last 12 months was reported by 130 participants (12.4%). Ninety-three percent also used amyl nitrite ("poppers"), 90% alcohol, 76.2% medication for erectile dysfunction, and 70.8% GHB/GBL in sexual settings in the last 12 months. All other substances are listed in **Table 1**.

**Table 1:** Sample characteristics and substance use.

	Methamphetamine group		No drug use for sex (NDUS)		p-value
		M (SD)		M (SD)	t-test
Age		34.5 (10.1)		37.6 (12.6)	.000
	N	%	N	%	χ^2^
Country of birth	88		145		.172
Germany		85.2		91.0	
Outside Germany		14.8		9.0	
Gender identity	130		173		.073
Male		100.0		97.1	
Trans man		0.0		2.9	
Sexual identity	129		168		.307
Gay/homosexual		93.8		89.9	
Bisexual		6.2		8.9	
Queer		0.0		1.2	
Level of education	81		134		.533
University or university of applied sciences entrance diploma		72.8		76.9	
General certificate of secondary education		16.0		14.2	
Certificate of secondary education		11.1		7.5	
No certificate		0		2.2	
Employment status	88		147		.013*
Full-time employed		65.9		62.6	
Part-time employed		5.7		8.2	
Unemployed		8.0		4.1	
Retired		8.0		3.4	
Student		3.4		17.0	
Other		9.1		4.8	
					
Monthly net income	87		148		.043*
Less than 1.000 Euros		17.2		24.9	
1.000–2.000 Euros		24.1		33.1	
2.000–3.000 Euros		25.3		23.6	
More than 3.000 Euros		33.2		18.3	
Substance use last 12 months **in a sexual setting**			Substance use last 12 months, not in sexual settings		
	n = 130	%	n = 170	%	
Amyl nitrite ("poppers")		93.8		0	
Medication for erectile dysfunction		76.2		0	
Gamma-hydroxybutyrate/gamma-butyrolactone (GHB/GBL)		70.8		0	
Amphetamines		62.3		0	
Alcohol		62.3		78.3	
Ecstasy		56.9		1.2	
Ketamine		53.8		0.6	
Cannabis		51.5		11.8	
Cocaine		46.9		0	
Mephedrone		40.8		0	
Opioid analgesic		5.4		4.1	
Heroin		0.8		0	

*p < .05.

The present analysis includes men who reported methamphetamine use in sexual settings in the last 12 months (n = 130; 8.2% of the sample) and men who reported no illicit drug use in sexual settings in the last 12 months (n = 170; 10.7% of the sample). For demographics of both groups, see **Table 1**.

### Mental Health Measures

The median PHQ-9 score was significantly higher in the methamphetamine group compared to the NDUS group. Eleven percent of participants in the methamphetamine group and 12.1% in the NDUS group had PHQ-9 scores above the cutoff for moderate depressive symptoms (see **Table 2**). There was no significant difference between the methamphetamine group and the NDUS group regarding GAD-7 scores. Five percent of participants in the methamphetamine group and 8.7% of participants in the NDUS group had a GAD-7 score of 10 or above, which indicates at least moderate levels of anxiety. There was no significant difference between the methamphetamine group and NDUS group regarding PHQ-15 scores. Thirteen percent of participants in the methamphetamine group and 10.6% in the NDUS group had a score of 10 or above, indicating at least moderate levels of clinically relevant somatization. In both groups together, 76.4% of participants had experienced at least one potentially traumatizing event according to the *DSM-5* life events scale, with a mean number of 1.86 potentially traumatizing events. There was no significant difference between the two groups in the number of traumatic events reported. In the methamphetamine group, 6.4% had a score of 3 or more on the PTSD primary care screener, indicating a possible diagnosis of PTSD. In the NDUS group, 12.9% were above the cutoff for PTSD, although the difference between the groups was not statistically significant (see **Table 2**).

**Table 2:** Mental health, infectious diseases, and biomedical HIV prevention.

Measure	Methamphetamine users			No Drug Use For Sex (NDUS)					
	N	Mdn= median	IQR IQR= interquartile range	N	Mdn= median	IQR IQR= interquartile range	Mann–Whitney U	p-value	Effect size (r)
PHQ-9	117	5.00	5.00	150	3.00	4.25	6,824.0	.002**	0.2
GAD-7	112	3.00	4.00	149	2.00	5.00	7,398.0	.113	0.1
PHQ-15	116	4.00	5.00	151	3.00	4.00	7,595.0	.061	0.1
Traumatic Events Lifetime	110	2.00	2.00	142	1.00	2.00	6,852.0	.087	0.1
	N	%		N	%		Chi^2^	p-value	Effect size (Cramer V)
PTSD screening positive	110	6.4		139	12.9		2.949	.086	0.1
PHQ-9 > 10	117	11.1		150	12.0		.051	.822	0.01
GAD-7 > 10	112	5.4		149	8.7		1.074	.300	0.064
PHQ-15 > 10	116	12.9		151	10.6		.345	.555	0.036
HIV positive	91	52.7		96	7.2		30.670	.000**	0.418
Hepatitis C infection	93	3.2		70	0		2.300	.129	0.119
Condomless anal intercourse	100	93		139	49.6		50.070	.000**	0.458
PrEP	43	53.8		83	7.2		29.126	.000**	0.481

PHQ-9, Patient Health Questionnaire (PHQ-D) subscale for depressive symptoms; GAD-7, PHQ-D subscale for generalized anxiety symptoms; PHQ-15, (PHQ-D) subscale for somatization symptoms; PTSD, posttraumatic stress disorder; PrEP, pre-exposure prophylaxis. Mdn, median; IQR, interquartile range. **p < .01.

### Infectious Diseases

Regarding HCV, 3.2% of the methamphetamine group reported that they were HCV positive. None of the NDUS group was positive for HCV. Fifty-three percent of the methamphetamine group reported being HIV positive, 42.9% were HIV negative, and 4.4% did not know their current HIV status. Compared to the NDUS group, participants in the methamphetamine group were significantly more likely to report being HIV positive (7.3% vs. 52.7%). All HIV-positive participants were taking HIV ART and reported having an undetectable viral load. Among HIV-negative men, a higher proportion of men in the methamphetamine group (53.8%) were currently taking PrEP than in the NDUS group (7.2%). Any condomless anal intercourse in the last 12 months was reported by a significantly higher proportion of men in the methamphetamine group (93.0%) than in the NDUS group (49.6%).

### Harm Reduction Practices

Participants who reported methamphetamine use for sex reported a range of drug- and sex-related harm reduction practices. The practices that men most commonly reported always doing were: drinking enough non-alcoholic beverages, making sure to get enough sleep after consumption, and having enough lube available at all times **Table 3**).

**Table 3:** Utilization of harm reduction strategies of methamphetamine-using participants.

Strategy	% always	% often	% sometimes	% never
**Safer use**				
Only using own needles and syringes (iv-users only)	79.6	20.4	–	–
Avoiding simultaneous use with tranquilizers	70.0	10.0	9.0	11.0
Using a new syringe and needle for every iv application (iv-users only)	75	19.2	5.8	–
Avoiding simultaneous use with alcohol	56.4	13.9	14.9	14.9
Bring own needles, syringes, and other utensils for consumption to parties (iv-users only)	64.2	13.2	11.3	11.3
Only using own tubule for nasal consumption	44.3	23.7	16.5	15.5
Inhaling methamphetamine instead of injecting it	34.0	15.5	22.7	27.8
Avoiding simultaneous use with other stimulants	30.4	20.6	19.6	29.4
Trying a small dose of a new stash to estimate the impact	28.0	21.0	12.0	39.0
Dispensing a dose over a longer stretch of time	25.8	27.8	28.9	17.5
Avoiding simultaneous use with medication for erectile dysfunction	21.0	9.0	20.0	50.0
Avoiding simultaneous use with poppers and medication for erectile dysfunction	14.6	10.4	27.1	47.9
Avoiding simultaneous use with poppers	12.1	12.1	22.2	53.5
**Health-related behavior**				
Drinking enough non-alcoholic beverages	63.1	23.3	11.7	1.9
Getting enough sleep after use	60.0	26.0	12.0	2.0
Avoiding consumption when feeling depressed or anxious	48.0	10.0	14.0	28.0
Using an alarm clock to remember HIV medication or PrEP	31.1	7.8	6.7	54.4
Eating sufficiently before consumptions	27.5	30.4	32.4	9.8
Eating regularly during consumption	9.9	22.8	35.6	31.7
**Sexual behavior**				
Having enough lube available at all times	62.2	22.4	11.2	4.1
Having no anal intercourse for half an hour after "booty bumping" (= substance application *via* intestinal mucosa)	28.1	15.7	20.2	36.0
Having condoms available at all times	13.4	8.2	17.5	60.8
Not having sex with more than one partner	3.1	7.2	22.7	67.0
**Frequency**				
Not consuming more than 2 days in a row	60.2	21.4	10.2	8.2
Only using on long weekends or special occasions	52.0	20.0	16.0	12.0
Not consuming more than once a month	36.4	27.3	25.3	11.1
Setting limits for quantity of consumption	36.0	24.0	26.0	14.0

iv, intravenous.

### Health Care and Psychosocial Support Service Utilization

Thirteen percent of men in the methamphetamine group and 4.1% in the NDUS group were seeing a psychotherapist at the time of the survey, while 6.9% in the methamphetamine group and 1.8% in the NDUS group were seeing a psychiatrist. Two percent (2.3%) of men in the methamphetamine group and 1.8% in the NDUS group were attending an outpatient alcohol and other drug counseling service at the time of the survey. In the methamphetamine group, the most common forms of engagement with the health care system were with a general practitioner (52.8%) or an infectious diseases specialist (35.2%). Participants also had contact with other psychosocial support services, including counseling for people living with HIV (17.4% methamphetamine group and 2.9% NDUS group) and LGBTIQ-specific counseling services (9.2% of participants in methamphetamine group and 0.6% in the NDUS group).

## Discussion

In this study, German MSM who used crystal methamphetamine commonly did so in the context of sexual activities. Poorer mental health status was observed among MSM who used methamphetamine than in the general male population. Men who used crystal methamphetamine for sex seemed to be aware of potential health risks associated with their substance use and utilized harm reduction strategies and biomedical HIV prevention strategies like PrEP and HIV treatment as prevention (TasP).

These results support previous research in a German sample of people who use psychostimulant that methamphetamine use for sex is an important motive of MSM (35). Polydrug use was commonly reported in this subset of methamphetamine-using German MSM. Nearly all participants used amyl nitrite in the previous 12 months, and more than two-thirds used erectile dysfunction medications. Amyl nitrite is commonly used as a muscle relaxant to facilitate receptive anal intercourse, and erectile dysfunction medications are often reported by MSM who used methamphetamine for sex as psychostimulant use is often associated with difficulties gaining and maintaining an erection (39). About half of the sample reported consumption of other substances that are commonly associated with chemsex (e.g., GHB/GBL, mephedrone, ketamine) (2).

Eleven percent in the methamphetamine group had a score on the PHQ-9 scale indicating at least moderate depressive symptoms. This is comparable to a sample of MSM in the UK (40), but considerably lower than in an Australian study among MSM. Here, nearly one-third of gay and bisexual men reported moderate depressive symptoms on the PHQ-9 scale (32). Regarding most mental health measures, there were no significant differences between men who used methamphetamine for sex and men who reported no drug use for sex. However, both groups of MSM reported consistently higher levels of depression, somatization (41), generalized anxiety (42), number of traumatic life events (43), and PTSD (44) compared to representative data among the general population of men in Germany. Crystal methamphetamine use does not seem to be the most contributing factor, given that both groups of MSM showed lower mental well-being.

In summary, the results underline that both groups of MSM, irrespective of substance use, were more likely to experience poor mental health than the male general population. According to the minority stress model (45), a minority status, like a non-heterosexual sexual orientation, has an impact on psychological well-being and can increase likelihood of experiencing problems with mental health and substance use.

Besides this, poorer mental health status among men who used methamphetamine in our study may be traced back to the fact that half of the sample was HIV positive. Since ART, HIV is a chronic condition similar to other chronic conditions [e.g., diabetes (46)], and living with HIV and other chronic conditions is associated with an increased likelihood of experiencing depression (47). Experiences of HIV-related stigma may also be a contributing factor to the higher rates of depression reported, which can negatively impact mental health and well-being (48).

The heightened proportion of men who live with HIV in the methamphetamine group is consistent with previous research (14, 15). All HIV-positive men in the sample were taking ART and self-reported an undetectable viral load. Successful treatment of HIV is an important contribution to prevent HIV transmission (TasP). Large-scale, prospective studies have shown no HIV transmission in serodiscordant couples when the viral load of the HIV-positive partner was suppressed sufficiently by ART (49). The latest addition to biomedical HIV prevention strategies is PrEP. PrEP refers to the use of HIV-antiretrovirals in HIV-negative people at high risk for HIV to prevent infection (50). In Germany, PrEP is only available on prescription and has been available at an affordable price since 2017. As a result of an initiative of a pharmacist in Cologne and negotiations with a pharmaceutical company to distribute a generic version of PrEP, it was available nationally for 50 Euros (about 55 USD) per month. Since September 2019, PrEP has been covered by health insurance free of charge for people at high risk of becoming infected with HIV. HIV-negative men who use methamphetamine seem to reflect that they may be at risk for HIV infection due to their substance use in sexual settings and therefore decide for PrEP. Under the influence of methamphetamines, other prevention strategies, like condom use, may be compromised (51). Nonetheless, PrEP and TasP do not prevent the acquisition of other STIs.

Half of the methamphetamine user sample injected drugs in sexual settings, which carries a risk for the transmission of blood-borne viruses like HCV, as well as HIV. Although the prevalence of HCV was 10 times higher than in the German general population (52), it was significantly lower than in other groups of people who inject drugs in Germany, which has been estimated at between 42% and 75% (53). The routine utilization of harm reduction strategies can help prevent HCV among people who inject drugs (54). In the German Chemsex Survey sample, men who use methamphetamine seem to be aware and well informed about various harm reduction strategies. Most participants used at least some harm reduction practices to prevent negative health outcomes related to methamphetamine use. Injecting substances carries the highest risk for negative health consequences. Among those who injected methamphetamine in sexual settings, harm reduction practices appeared to be well established. More than two-thirds of men who injected methamphetamine stated that they always used their own needle and syringe and used a new needle and syringe every time they injected. There has been very little research published on the harm reduction practices of MSM. In a Canadian study, harm reduction strategies with focus on safety when injecting drugs were most common (55). Other strategies refer to restrictions of frequency or maintaining a healthy lifestyle, such as eating regularly, getting enough sleep, and staying hydrated (55). Avoidance of polydrug use is another important harm reduction practice, given the increased risk of overdose and other negative consequence of combining drugs in the same session. Half of the sample stated that they never refrain from simultaneous use of methamphetamine and amyl nitrite or erectile dysfunction medications. One-third reported never avoiding using other stimulants at the same time as methamphetamine. Combined use of erectile dysfunction medications and alcohol, other recreational drugs, and especially amyl nitrite, increases risks for potentially fatal cardiovascular events and other serious drug interactions (56). Given that polydrug use was common in this sample and in other studies of MSM (57), there is some potential for improvement of applying this harm reduction strategy.

About one in five men of the sample currently consults a psychotherapist or psychiatrist. This is a good fit to the proportion of men who report mental health problems in the sample, so mental health care service utilization seems to be high. Only few men seek support from alcohol or drug treatment facilities. Perhaps, they do not need such treatment since they do not have any substance-related problems or disorders. Other reasons could be that there are only very few target group–specific services for MSM who use drugs for sex or that they fear rejection or stigmatization by service staff because of their sexual orientation. Future work could have a further look at what type of counseling or treatment services MSM who use drugs for sex need and where those services should be located. An integration of sexual health, LGBTIQ counseling, and drug treatment services would be helpful to exchange expertise and improve care for support-seeking MSM who use drugs for sex.

### Limitations

About one in five participants reported lifetime methamphetamine use, which is a considerably higher prevalence than in the German male general population. The German Chemsex Survey was not designed to determine prevalence rates for methamphetamine use among MSM, but to recruit a sample of MSM who report substance use, and was advertised accordingly. The results should thus be interpreted with this in mind, and may not be generalizable to all MSM in Germany. The inclusion of a self-selected convenience sample may contribute to bias, with overestimation of substance use in sexual settings and mental health problems. In addition, the sample had a high socioeconomic and educational status, clearly above the average in the German male general population. Moreover, the sample was not diverse as very few trans men or men born outside of Germany participated. The survey was only available in German, so men with insufficient German language skills would have been discouraged from participating. The high number of HIV-positive participants may be due to the recruitment sources as the survey was promoted *via* community-based organizations that provide services for people living with HIV. Another obvious limitation is the high rate of attrition, most likely due to the large number of items. Despite these limitations, the study provides some relevant findings on MSM who use substances in sexual settings, regarding mental health, biomedical HIV prevention, and harm reduction strategies.

## Data Availability Statement

The datasets generated for this study are available on request to the corresponding author.

## Ethics Statement

The studies involving human participants were reviewed and approved by the Ethics Committee of the Medical Department of the University of Duisburg-Essen. The patients/participants provided their written informed consent to participate in this study.

## Author Contributions

HS: study conceptualization, data analysis, article writing. TL: article writing, language editing. AB: data analysis and literature search. DS: study conceptualization, editing article. TK: consulting data analysis. NS: editing article. DD: study conceptualization, editing article.

## Conflict of Interest

NS received honoraria for several activities (advisory boards, lectures, manuscripts) by AbbVie, Hexal, Janssen-Cilag, MSD, Medice, Mundipharma, Reckitt-Benckiser/Indivior, and Sanofi-Aventis. During the last 3 years, he participated in clinical trials financed by the pharmaceutical industry.

The remaining authors declare that the research was conducted in the absence of any commercial or financial relationships that could be construed as a potential conflict of interest.
